# Heartworm (*Dirofilaria immitis*) in carnivores kept in zoos in Texas, USA: risk perception, practices, and antigen detection

**DOI:** 10.1186/s13071-023-05750-z

**Published:** 2023-04-28

**Authors:** Kaitlyn E. Upton, Christine M. Budke, Guilherme G. Verocai

**Affiliations:** 1 Department of Veterinary Pathobiology, School of Veterinary Medicine and Biomedical Sciences, Texas A&M University, College Station, TX USA; 2 Department of Veterinary Integrated Biosciences, School of Veterinary Medicine and Biomedical Sciences, Texas A&M University, College Station, TX USA

**Keywords:** Asian small-clawed otter, Carnivora, Chemoprophylaxis, Heartworm disease, Immunodiagnostics, Zoological medicine, Prevention

## Abstract

**Background:**

*Dirofilaria immitis* is the causative agent of heartworm disease in wild and domestic canids, felids, and mustelids. Recent studies demonstrate that additional families in the order Carnivora are also susceptible to infection. Therefore, the objectives of this study were to (1) better understand current practices surrounding heartworm prevention and diagnostics in zoological facilities located in the state of Texas, USA, and (2) assess archival serum samples of carnivores kept in these facilities for the presence *D. immitis* antigen and/or antibody.

**Methods:**

A questionnaire was completed by veterinarians or veterinary technicians representing 10 zoological facilities across Texas. This questionnaire was designed at the taxonomic family level, encompassing the 12 terrestrial carnivore families Ailuridae, Canidae, Eupleridae, Felidae, Herpestidae, Hyaenidae, Mephitidae, Mustelidae, Prionodontidae, Procyonidae, Ursidae, and Viverridae. The second objective was achieved with the use of archival serum samples made available by six zoo facilities.

**Results:**

Risk perception varied across facilities for every family, including among species belonging to Canidae. All facilities used monthly heartworm prevention in canids and felids, with more variation existing in the other families. The use of diagnostic testing and type and route of administration of preventive varied by facility, with oral ivermectin the most commonly used preventive. A total of 217 archival serum samples, belonging to 211 individual animals encompassing 11 families and 39 species, were tested with a commercial heartworm antigen ELISA test, pre- and post-immune-complex dissociation. A subset of samples was also assessed for the presence of feline anti-heartworm antibodies using a commercial ELISA test. Two animals, both of which were Asian small-clawed otters from the same facility, had antigen detected (0.95%).

**Conclusions:**

This study demonstrates that while the zoo veterinary community is aware of the risk and health impact of heartworm disease in canids and felids, there is still a great deal of uncertainty regarding the risks and ideal strategies for prevention in other carnivore families. The low proportion of antigen detection may serve as a baseline for future prevalence studies across the southern United States, where there is an emerging concern of macrocyclic lactone resistance in heartworm.

**Graphical Abstract:**

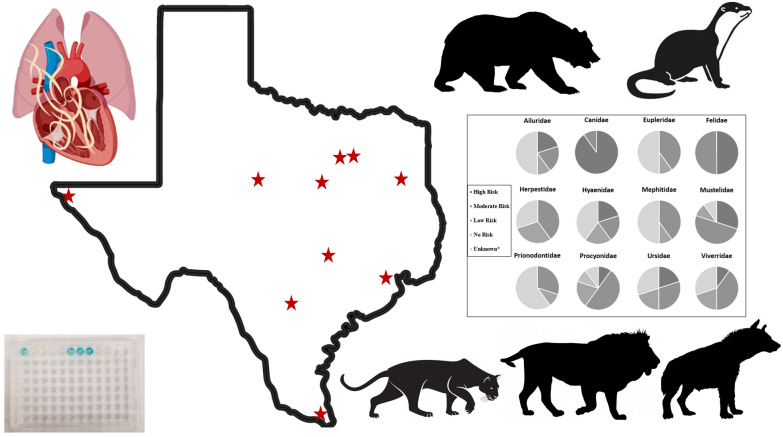

**Supplementary Information:**

The online version contains supplementary material available at 10.1186/s13071-023-05750-z.

## Background

Heartworm (*Dirofilaria immitis*) is one of the most important parasites of companion animals in the United States of America (USA) and is often associated with serious or fatal disease [1]. This parasite is transmitted by Culicidae mosquitoes and is highly prevalent across the southern states [2, 3]. Year-round prevention through the use of macrocyclic-lactone (ML) products and annual testing are recommended for pets, including domestic dogs, domestic cats, and ferrets [4]. Despite the availability of effective products and sensitive diagnostic tests, there are challenges to protecting pets, including lower than desired compliance and the emergence of anthelmintic resistance to preventive ML drugs. Carnivores in zoos located in heartworm-endemic areas should also be considered at risk of infection. The inherent phylogenetic diversity of carnivores and the lack of labeled products for such species hamper the implementation of standardized recommendations and practices. Such challenges may explain reports of heartworm infection in carnivore species that are often not considered at high risk of heartworm infection.

In addition to companion animals, heartworm infection or associated diseases have been reported from wild carnivores within the families Canidae and Felidae, including coyotes, wolves, golden jackals, foxes [5–10], lynxes [11], an ocelot [12], an oncilla [13], various leopard species [14–16], a lion [17], and a black-footed cat [18]. However, infection with *D.* *immitis* is not limited to species within these two carnivore families as there have been reports in various other species, including raccoons [19], various species of otters [20–22], and both brown and black bears [23, 24]. Most knowledge surrounding heartworm disease comes from research in domestic dog and cat species, while little is known about the disease in the wild members of the order Carnivora.

While heartworm prevention and diagnostics may be routinely performed for most carnivore species maintained in zoological collections accredited by the Association of Zoos and Aquariums (AZA) and/or the Zoological Association of America (ZAA), some carnivore families may be overlooked as susceptible to infection. In addition, even when heartworm prevention is administered, most protocols are empirical, and extrapolated from recommendations for other carnivore species. In many instances, preventive treatment may have less than desirable efficacy. Therefore, there is a need to assess carnivores belonging to those families generally seen as susceptible (i.e., Canidae, Felidae, Mustelidae), but also those belonging to families with scarce to no reports of heartworm infection, living in a heartworm-endemic area such as Texas [25, 26].

The objectives of this study were to (1) better understand current practices surrounding heartworm prevention and diagnostics in zoological facilities located in the state of Texas, USA, and (2) assess archival serum samples of carnivores kept in these facilities for the presence of heartworm antigen and/or antibody.

## Methods

### Questionnaire on risk perception and management practices

Facilities targeted for this questionnaire were those in the state of Texas, USA, holding accreditation with either the AZA or ZAA, or both entities. Aquarium and hoof stock-exclusive facilities were not included. All such qualifying facilities, 15 in total, were contacted via email, phone, or both methods depending on available contact information. A questionnaire was created using Google Forms, with closed-end questions as well as an additional option that allowed for a free-response answer to be added. The questionnaire was distributed via email, which included a link to the Google Form as well as the questionnaire as PDF and Microsoft Word document attachments, allowing for online completion or email submission of a handwritten or typed version. The questionnaire was distributed from August through October of 2020, and results were accepted through February of 2021.

The questionnaire was created to assess basic information about the facility, heartworm risk perception, use and type of heartworm prevention, method and frequency of testing for heartworm, and history of *D. immitis* infection in the facility. Questions related to the 12 terrestrial families within the order Carnivora, Ailuridae, Canidae, Eupleridae, Felidae, Herpestidae, Hyaenidae, Mephitidae, Mustelidae, Prionodontidae, Procyonidae, Ursidae, and Viverridae were included in the questionnaire, while pinnipeds were excluded. The questionnaire was designed to be completed by veterinarians or veterinary technicians who were familiar with their facilities’ preventive medicine protocols.

### Acquisition and laboratory testing of biological samples

For the second part of this study, archival serum samples were obtained from facilities that participated in the first part of the study by completing the questionnaire. All respondents were contacted about continuing to participate via the contribution of archival serum samples from animals in any of the 12 families that were the focus of the questionnaire. Six facilities contributed samples. These samples had collection dates ranging from 2012 to 2021, and the most recent available sample from each animal was requested. Samples were stored at their respective facilities between −80 and −20 °C. All samples underwent antigen testing, both pre- and post-immune complex dissociation (ICD), via the PetChek^®^ Heartworm PF Antigen Test (IDEXX Laboratories, Inc., Westbrook, ME, USA). ICD was achieved according to a previously described heat-treatment protocol [27]. Thirteen low-volume samples were diluted 1:1 with 0.1 M ethylenediaminetetraacetic acid (EDTA) [27, 28], and a known positive canine sample was used as a positive control for the dilution. Antibody testing was performed on felid samples via the Solo Step^®^ Feline Heartworm Antibody Test (Heska, Loveland, CO, USA). Sixty-two felids were reported by their facilities as being tested annually for heartworm antibodies and, therefore, were excluded from testing. Select mustelid samples were also tested with the same feline antibody test after the antigen results were reported.

### Statistical analysis

A unipolar second-generation Potential for Conflict Index (PCI_2_) with a distance function of 3 (D_3_) and a power function of 1 (P_1_) was calculated to evaluate agreement between heartworm risk perception responses within each family [29]. Responses of “Unknown” (including no response) were excluded from analysis. The following Likert-type scale scoring for the computation of means was used for each response: no risk = 1, low risk = 2, moderate risk = 3, high risk = 4. Interpretation of the PCI_2_ ranges from a score of 0 (minimum potential for conflict/more agreement) to a score of 1 (maximum potential for conflict/less agreement).

## Results

### Questionnaire

**Fig. 1:**
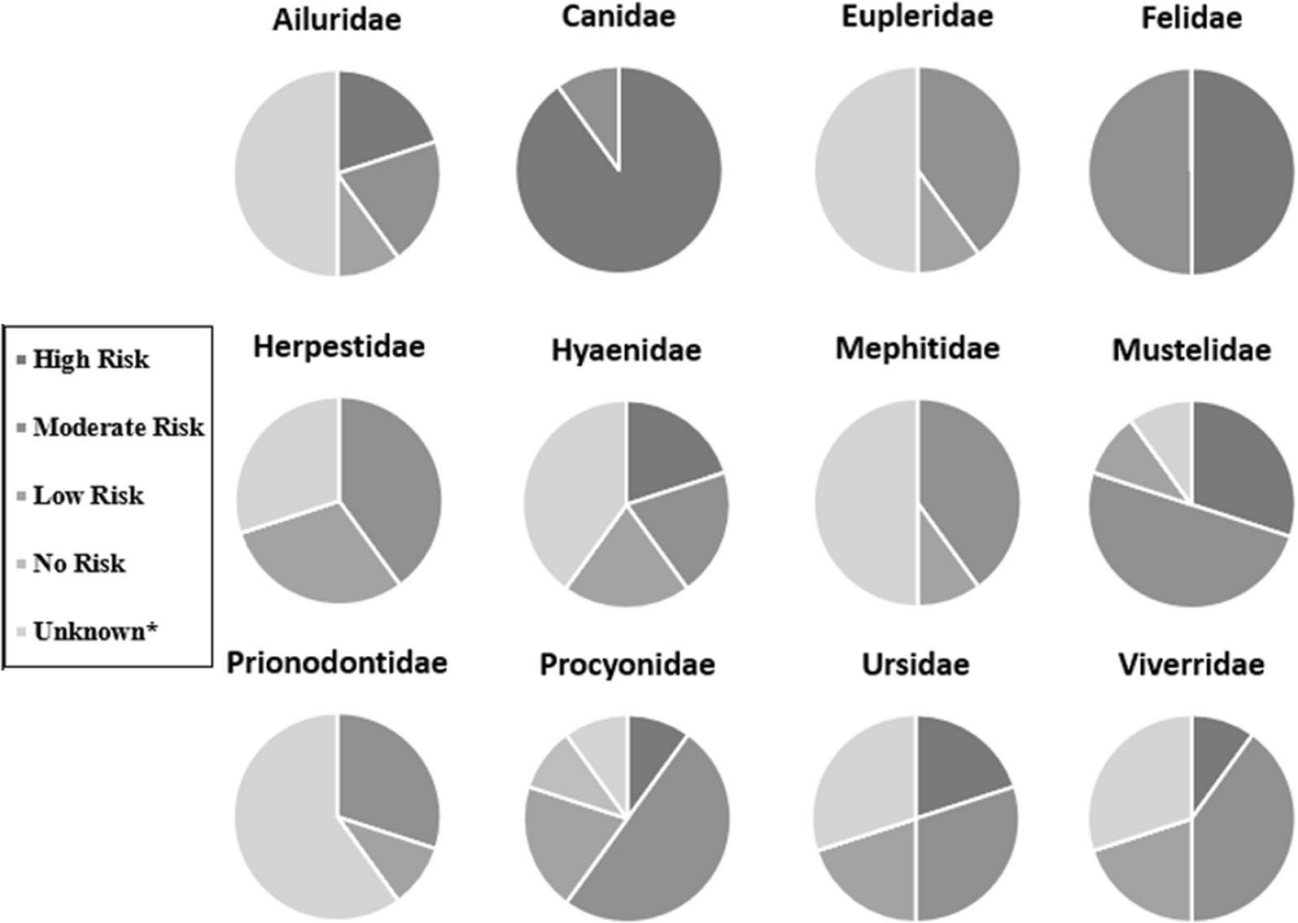
Risk perception of *D. immitis* by carnivore family

Ten facilities (randomly assigned letters A–J) submitted completed questionnaires (10/15; 66.67% response rate). Seven questionnaires were completed by veterinarians and three were completed by veterinary technicians. The facilities ranged in size from 501 to over 4000 animals in their collections, in which carnivores comprised from less than 1% to 30% of animals. Two facilities reported having at least one animal previously diagnosed with heartworm disease, but no timeframe was specified for these cases. One additional facility noted that a guard dog that lived on the property but was not considered part of the collection had been diagnosed with heartworm disease and undergone adulticidal treatment. Respondents from all 10 facilities agreed that heartworm is a problem in their city/county. Risk perception data were consistent across facilities for the family Canidae, with nine facilities (90%) considering members of this family at high risk and one facility (10%) considering members of this family at moderate risk. As for the family Felidae, five facilities (50%) considered members of this family at high risk, and the other five (50%) considered members of this family at moderate risk (50%). There was much greater variation in the perceived risk for the remaining 10 families (Fig. 1, Table 1).

**Table 1:** Risk perception and second-generation Potential for Conflict Index (PCI_2_) and mean Likert scores (1 = no risk, 2 = low risk, 3 = moderate risk, 4 = high risk) of *D. immitis* by zoo facility

	Ailuridae	Canidae	Eupleridae	Felidae	Herpestidae	Hyaenidae	Mephitidae	Mustelidae	Prionodontidae	Procyonidae	Ursidae	Viverridae
Facility A	U	H	U	M	U	U	U	U	U	U	U	U
Facility B	M	H	M	M	M	M	M	M	M	M	M	M
Facility C	U	H	U	H	U	U	U	H	U	L	U	M
Facility D	L	H	L	M	L	L	L	L	L	L	L	L
Facility E	H	M	M	M	L	M	M	M	M	M	M	M
Facility F	U	H	U	M	M	M	U	U	M	U	M	M
Facility G	U	H	U	H	U	U	U	H	U	H	U	U
Facility H	U	H	U	H	L	L	U	M	U	N	L	L
Facility I	H	H	M	H	M	H	M	H	U	M	H	M
Facility J	M	H	M	H	M	H	M	M	M	M	H	H
PCI_2_	0.56	0.12	0.22	0.33	0.33	0.56	0.22	0.46	0.22	0.60	0.56	0.40
Mean Score	3.00	3.90	2.80	3.50	2.57	3.00	2.80	3.25	2.80	2.63	3.00	2.88

*H* high risk, *M* moderate risk, *L* low risk, *N* no risk, *U* unknown risk (including no response)

All 10 facilities reported the use of heartworm prevention in many of their carnivores (Table 2), including all canids and felids. Five of the 10 participating facilities had 100% of their terrestrial carnivores on some form of heartworm prevention. Ailurids, euplerids, and hyaenids all received prevention in the facilities in which they were represented. Ursids and viverrids received prevention in all but one facility, which reported no prevention use in either family. There was more variation in the remaining families. Mephitids received prevention in half of the facilities (2/4) in which they were represented, while herpestids received prevention in four of six facilities. Procyonids, represented in seven facilities, received prevention in four facilities, while a fifth facility reported that “most” of the procyonids in the collection were receiving prevention. Two facilities did not use prevention in procyonids. All mustelids received prevention in seven facilities, with an eighth facility reporting that “some” of the mustelids in the collection received prevention.

**Table 2:** Heartworm prevention use by facility

	Ailuridae	Canidae	Eupleridae	Felidae	Herpestidae	Hyaenidae	Mephitidae	Mustelidae	Procyonidae	Ursidae	Viverridae
Facility A		P		P							
Facility B		P	P	P	P	P	P	P	P	P	P
Facility C		P		P				P	N		P
Facility D		P		P	N	P	N	P	P	P	
Facility E	P	P	P	P	N		P	P		P	
Facility F		P		P	P			P		P	
Facility G		P		P				N	M	N	N
Facility H		P		P	P			P	P	P	
Facility I		P		P	P			P	P	P	
Facility J		P	P	P			N	S	N		

Blank spaces indicate a family that is not represented in the facility

*P* Family represented in facility and all animals on prevention, *M* family represented in facility and MOST animals are on prevention, *S* family represented in facility and SOME animals are on prevention, *N* family represented in facility but not on prevention

Euplerids received prevention monthly in all facilities in which they were represented, with some euplerids in one of those facilities receiving prevention every 2 months. All other families receiving heartworm prevention in all facilities were given prevention monthly. The most common prevention used across all facilities and families was ivermectin labeled for use in cattle (Table 3). Selamectin, a topical avermectin commonly used in dogs and cats for the prevention of heartworm disease, was used by four facilities in five families: Felidae, Herpestidae, Mephitidae, Mustelidae, and Procyonidae. Milbemycin oxime, an oral milbemycin commonly used in dogs and cats for the prevention of heartworm disease, was used by four facilities in two families: Canidae and Procyonidae. All four of these facilities used this product in canids, with one facility also using it in procyonids. Prevention products were administered either topically or orally, with oral medications typically put in meat or other food (Table 4).

**Table 3:** Heartworm prevention products used in each family

Family	Prevention product(s)	Facilities
Ailuridae	Ivermectin	E
Canidae	Ivermectin	B, C, D, E, F, G, H, I, J
	Milbemycin oxime	A, E, G, J
Eupleridae	Ivermectin	B, E, J
	Moxidectin	E
Felidae	Ivermectin	ALL (100%)
	Selamectin	F
Herpestidae	Ivermectin	B, F, I
	Selamectin	H
Hyaenidae	Ivermectin	B, D
Mephitidae	Ivermectin	B
	Selamectin	E
Mustelidae	Ivermectin	B, C, D, E, F, J
	Selamectin	H
Procyonidae	Ivermectin	B, D, E, I
	Milbemycin oxime	G
	Selamectin	G
	Other^a^	H
Ursidae	Ivermectin	B, D, E, F, H, I
Viverridae	Ivermectin	B, C

^a^Zoo personnel mentioned having attempted to offer preventive products containing ivermectin, milbemycin oxime, selamectin, and moxidectin products to a raccoon

**Table 4:** Route of administration for heartworm prevention by facility

	Ailuridae	Canidae	Eupleridae	Felidae	Herpestidae	Hyaenidae	Mephitidae	Mustelidae	Procyonidae	Ursidae	Viverridae
Facility A		M		M							
Facility B		M	M	M	M	M	M	M	M	M	M
Facility C		M		M				M, O	N		M, O
Facility D		M		M	N	M	N	M	M	M	
Facility E	O	M	M, T	M	N		T	M		M, O	
Facility F		M		M, T	M			M, T		M	
Facility G		M		M				N	T	N	N
Facility H		M		M	T			U	P	M	
Facility I		M		M	M			U	M	O	
Facility J		M	M, O	M			N	M, O	N		

*P* by mouth, *M* by mouth, mixed in meat, *O* by mouth, mixed in other food items, *T* topical or pour-on, *U* unspecified, *N* no prevention

Blank spaces indicate a family that is not represented in the facility

Routine diagnostic testing for *D. immitis* was performed in most facilities before and/or after transportation, if an animal was ill, during routine exams every 1–3 years, or opportunistically (Table 5). One facility reported testing all carnivores yearly with a canine *D. immitis* antigen test. The canine antigen test was used in all facilities and was the most common diagnostic test reported (Table 6). Feline antigen tests were used in 8/10 facilities for felids and were also used in euplerids, herpestids, hyaenids, mephitids, mustelids, procyonids, and ursids in varying facilities. Feline antibody testing was used for felids in five facilities as well as mustelids in two of those facilities and ursids in one facility. Modified Knott’s testing was used in one facility in canids, felids, procyonids, and ursids.

**Table 5:** Frequency of diagnostic testing for *D. immitis* by facility

	Ailuridae	Canidae	Eupleridae	Felidae	Herpestidae	Hyaenidae	Mephitidae	Mustelidae	Procyonidae	Ursidae	Viverridae
Facility A		Y		R							
Facility B		Y	Y	Y	Y	Y	Y	Y	Y	Y	Y
Facility C		R		R				R	N		R
Facility D		T, R, O		T, R, O	T, R, O	T, R, O	U	T, R, O	T	T, R, O	
Facility E	T, R	T, R	T, R	T, R	T, R		T, R	T, R		T, R	
Facility F		S, T		S, T	S			S, T		S, T	
Facility G		S		S				N	N	N	N
Facility H		Y, S, T		Y, S, T	S, T			S, T	S, T	S, T	
Facility I		Y		R	O			N	Y	R	
Facility J		R	T	R			N	R	N		

*Y* yearly, *R* during routine physical exams every 1–3 years, *T* before and/or after transport to/from another facility, *S* when ill or showing related clinical signs, *O* opportunistically, *U* unspecified, *N* not performed

Blank spaces indicate a family that is not represented in the facility

**Table 6:** Routine diagnostic testing methods for *D. immitis* by facility

	Ailuridae	Canidae	Eupleridae	Felidae	Herpestidae	Hyaenidae	Mephitidae	Mustelidae	Procyonidae	Ursidae	Viverridae
Facility A		CT		FY							
Facility B		CT	CT	CT	CT	CT	CT	CT	CT	CT	CT
Facility C		CT		FT				FT, FY	N		U
Facility D		CT		FT, FY	FT	FT	FT	FT	FT	FT	
Facility E	CT	CT	FT	FT	CT		CT	CT		CT	
Facility F		CT, FY		FT, FY	N			FT, FY		CT, FY	
Facility G		CT		FT				N	N	N	N
Facility H		CT		FT	CT			CT	CT	CT	
Facility I		CT, K		CT, FT, FY, K	CT			CT	CT, FT, K	CT, K	
Facility J		CT	U	FT, FY			N	T, Y	N		

*CT* canine antigen test, *FT* feline antigen test, *T* non-species-specific antigen test, *FY* feline antibody test, *Y* non-species-specific antibody test, *K* modified Knott’s test, *W* wet mount, direct smear, *U* unspecified or other, *N* not performed

Blank spaces indicate a family that is not represented in the facility

### Biological sample testing

In total, 217 samples from 211 animals were received from six zoological facilities (A–F). Replicate samples were inadvertently contributed for six animals. Facility A contributed 57 samples from 56 individuals, Facility B contributed 31 samples, Facility C contributed 24 samples from 19 individuals, Facility D contributed 36 samples, Facility E contributed 68 samples, and Facility F contributed one serum sample from a recent necropsy specimen. Thirty-nine distinct species from 11 families were represented (Table 7). Three and four positive samples were detected on pre- and post-ICD antigen testing, respectively. The four positive samples were from two Asian small-clawed otters (*Aonyx cinereus*) in the same facility (Facility C). Neither otter had any clinical signs associated with *D. immitis* at the time of blood sample collection. Coincidentally, these two individuals both had replicate samples from 2018 and 2021 and 2019 and 2021, respectively (Table 8). Overall, 0.95% of animals (2/211) were antigen-positive on both pre- and post-ICD. On pre-ICD, 1.38% of samples (3/217) were positive and on post-ICD, 1.84% of samples (4/217) were antigen-positive. All felid samples tested (51/113) were negative for feline *D. immitis* antibodies. Additionally, the four positive otter samples were also negative for feline *D. immitis* antibodies. The proportion of antigen detection of *D. immitis* in animals for which samples were submitted from Facility C was 10.53% (2/19), while the overall proportion of antigen detection in this study for animals sampled from the family Mustelidae was 15.38% (2/13). For complete antigen testing results and sample distribution by family, species, and facility, see Additional file 1: Table S1.

**Table 7:** Samples tested for heartworm per carnivore species and reports in the literature

Family and species	Common name	Samples	Reports of *D. immitis*^b^
Ailuridae		2	
*Ailurus fulgens*	Red panda	2	[41]
Canidae		37	
*Canis latrans*	Coyote	1	[5–7, 9, 39, 40]
*Canis lupus baileyi*	Mexican gray wolf	5	[42]^a^
*Canis lupus dingo*	Dingo	2	[43]
*Canis rufus*	Red wolf	3	[39]^a^
*Chrysocyon brachyurus*	Maned wolf	7	[44]
*Lycaon pictus*	African painted dog	10	
*Otocyon megalotis*	Bat-eared fox	1	
*Speothos venaticus*	Bush dog	3	
*Urocyon cinereoargenteus*	Grey fox	1	[45]
*Vulpes velox*	Swift fox	3	
*Vulpes zerda*	Fennec fox	1	[10]
Eupleridae		3	
*Cryptoprocta ferox*	Fossa	3	
Felidae		113	
*Acinonyx jubatus*	Cheetah	53	
*Caracal caracal*	Caracal	3	
*Leopardus pardalis*	Ocelot	5	[12]
*Leptailurus serval*	Serval	1	
*Lynx rufus*	Bobcat	3	
*Neofelis nebulosa*	Clouded leopard	5	[16]
*Panthera leo krugeri*	African lion	15	[17]^a^
*Panthera onca*	Jaguar	10	[46]
*Panthera pardus*	Leopard	1	[15]
*Panthera tigris*	White tiger	1	[47]
*Panthera tigris jacksoni*	Malayan tiger	5	[47, 48]^a^
*Panthera tigris sumatrae*	Sumatran tiger	7	[47, 48]^a^
*Prionailurus viverrinus*	Fishing cat	1	
*Puma concolor*	Cougar	3	[49]
Herpestidae		17	
*Helogale parvula*	Common dwarf mongoose	1	
*Mungos mungo*	Banded mongoose	4	
*Suricata suricatta*	Slender-tailed meerkat	12	[50]
Hyaenidae		5	
*Crocuta crocuta*	Spotted hyena	2	
*Hyaena hyaena*	Striped hyena	3	
Mephitidae		2	
*Mephitis mephitis*	Striped skunk	2	
Mustelidae		17	
*Aonyx cinereus*	Asian small-clawed otter	12	[22]
*Lontra canadensis*	North American river otter	5	[20]
Procyonidae		14	
*Bassariscus astutus*	Ringtail	5	
*Nasua narica*	White-nosed coati	5	Diagnostic lab records
*Potos flavus*	Kinkajou	2	
*Procyon lotor*	Raccoon	2	[19]
Ursidae		6	
*Tremarctos ornatus*	Spectacled bear	2	
*Ursus americanus*	American black bear	3	[23]
*Ursus americanus luteolus*	Louisiana black bear	1	[23]^a^
Viverridae		1	
*Arctictis binturong*	Binturong	1	

^a^Represents a case of *D. immitis* in a different or unspecified subspecies

^b^This is not an exhaustive list and is likely an underrepresentation as many cases of *D. immitis* in exotic and wildlife species go undiagnosed or are not reported in the peer-reviewed literature

**Table 8:** Results from various testing methods for positive Asian small-clawed otter (*Aonyx cinereus*) samples

Sample	Pre-ICD antigen test	Post-ICD antigen test	Feline antibody test
Otter 1 - 2018	Negative	Positive	Negative
Otter 1 - 2021^a^	Positive	Positive	Negative
Otter 2 - 2019	Positive	Positive	Negative
Otter 2 - 2021	Positive	Positive	Negative

^a^EDTA diluted sample

### Statistical analysis

**Fig. 2:**
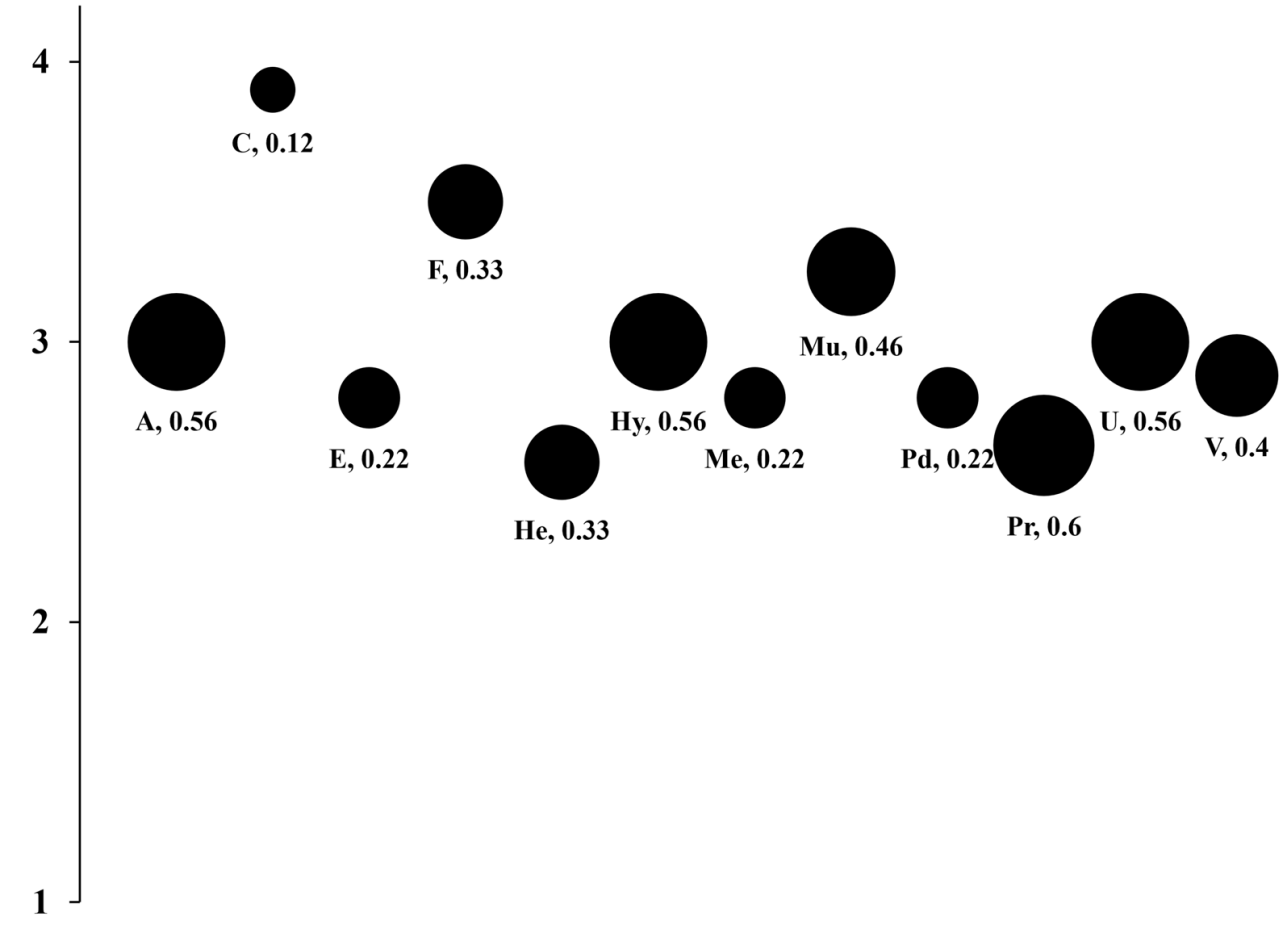
Agreement between facilities on risk perception of *D. immitis* by carnivore family. Numbers listed for each bubble and the size of the bubble represent the PCI_2_ with larger bubbles representing less agreement. The middle of each bubble represents the mean risk perception for each family (1 = no risk, 2 = low risk, 3 = moderate risk, 4 = high risk). *A* Ailuridae, *C* Canidae, *E* Eupleridae, *F* Felidae, *He* Herpestidae, *Hy* Hyaenidae, *Me* Mephitidae, *Mu* Mustelidae; Pd: Prionodontidae, *Pr* Procyonidae, *U* Ursidae, *V* Viverridae

Mean scores for heartworm risk perception, at the family level, ranged from 2.57 for Herpestidae to 3.9 for Canidae. The PCI_2_ demonstrated the greatest agreement between responses for Canidae at 0.12, while the largest disparity in responses was seen for Procyonidae at 0.60 (Table 1, Fig. 2). Canidae and Felidae were the only families with 100% of respondents providing scores.

## Discussion

The purpose of this study was to better understand current practices in zoological facilities in Texas surrounding heartworm risk perception, prevention, and diagnostics, and to assess the proportion of archived serum samples from carnivores housed in these facilities that were *D. immitis*-positive. This study demonstrated the variation in risk perception and management strategies in Texas zoological facilities for *D. immitis*. Even greater variation likely exists with responses such as “Some” or “Most” demonstrating variation that exists at the species or individual animal level. Additionally, since every family was not represented at every zoo, it is possible that more variation would exist if every facility had all families represented. Because data beyond risk perception were only taken into consideration for species that were represented in the collection at the time of questionnaire completion, it is impossible to assess the protocols for unrepresented families. The method of questionnaire completion may have also added to the variation as participants completing the form online could not skip questions or provide customized answers to certain questions. Canids, felids, and mustelids had the highest mean risk perception scores, which is not surprising given that these are the animals most often diagnosed with *D. immitis*. Canidae also had the lowest PCI_2_ value (0.12), demonstrating the greatest agreement of any family across all respondents. Eupleridae, Mephitidae, and Prionodontidae had low PCI_2_ values, indicating high levels of agreement on perceived risk between raters. However, there were only five respondents providing scores for each family, with the other five respondents choosing “Unknown” or giving no response. Procyonidae had the highest PCI_2_ value (0.60) demonstrating a lack of agreement on perceived risk amongst respondents. This variability may be due that most procyonid species kept in these zoos were native to areas of Texas and the United States (e.g., raccoons, ringtails, coati) and are no conservation concern, but it also could be dependent on which species was/were envisioned by the respondent when asked about this family.

As demonstrated by the questionnaire results (Table 6), many facilities rely on antigen testing exclusively. In domestic dogs and cats, it has been established that false-negative results can occur with antigen testing alone and it is recommended by the American Heartworm Society (AHS) to test for the presence of microfilaria in combination with yearly antigen testing [4]. The use of ICD in antigen testing is also recommended to increase diagnostic reliability and to aid in the detection of low worm burdens, all male infections, or early infections, especially in cats [30, 31]. Multiple facilities in this study utilized feline antibody testing in felids as well as other families. In all but one facility, the antibody tests were used in combination with antigen tests. The feline antibody test is designed to detect a feline antibody from the domestic cat, and while other felid species may have varying levels of conserved antibodies, this may not be a reliable method for the detection of antibodies against *D. immitis* in other families. The four positive otter samples in this study were all tested for feline antibodies and were all negative despite positive antigens in three out of four prior to ICD and all four post-ICD. This further demonstrates that the use of the feline antibody test likely has limited utility outside of the family Felidae.

This study and others in zoo and wildlife species often rely on opportunistically collected samples, which does not allow for accurate determination of prevalence. This study potentially biases samples towards animals that were ill and, therefore, had blood samples collected more regularly, those that were more easily handleable, or physically larger species or individuals as these animals can typically provide larger blood samples. It is important to assess what is considered the ideal management strategy as well as what is practical in a zoo setting with animals that cannot be handled as readily as pets. This presents a unique challenge in creating prevention and diagnostic protocols for both zoo and wildlife species. The AZA has published care manuals for various species and families, and there are varying recommendations on heartworm testing and prevention. While monthly prevention in endemic areas is commonly recommended, typically with ivermectin, the dosages are variable or left to the discretion of the veterinarian caring for the animal [32–34].

To the authors’ knowledge, there are no reports of pharmacokinetic or pharmacodynamic data on these MLs in non-domestic carnivores. However, doses are often extrapolated from studies in companion animals. Monthly ivermectin at a minimum dosage of 0.024 mg/kg has been proven efficacious against the larval stage of *D. immitis* in cats [35]. The AZA Lion (*Panthera leo*) Care Manual follows this guideline by recommending 0.025 mg/kg of oral ivermectin monthly. However, the AZA Jaguar (*Panthera onca*) Care Manual recommends 0.2 mg/kg, further demonstrating variability even within recommendations for closely related species and the need for pharmacokinetic data in these species [32, 33]. A report of doramectin toxicity in lions further reinforces the importance of appropriate, species-specific dosing of avermectins in carnivores [36].

Endemicity and prevalence of heartworm in companion animals in Texas is high. According to the Companion Animal Parasite Council (CAPC), the prevalence of heartworms in client-owned domestic dogs in the state of Texas, considering the last 5 years with data available (2017–2021), ranged from 2.87 to 3.45% [26]. Another study published in early 2019 tested shelter dogs across Texas that were not on heartworm prevention and found prevalence of 16% [37]. According to yearly CAPC data from 2017 to 2021, the percentage of cats in Texas testing antigen-positive ranged from 1.45 to 3.85%, and cats testing antibody-positive ranged from 0.53 to 1.32% [26]. In addition to the existing risk of heartworm infection in an endemic area, susceptibility of various carnivore species is supported by epidemiological studies in wildlife and case reports in wild and captive species that occur in Texas [5, 7, 10, 12, 38–40]. Moreover, many of the species represented in our sampling have been reported infected with heartworm or had antigen detected via commercial enzyme-linked immunosorbent assay (ELISA) kits (Table 7). Despite these factors, antigen detection in this study was low, suggesting that prevention methods being employed are working.

Antigen testing was utilized both pre-ICD and post-ICD (via heat treatment) in order to increase the sensitivity of antigen detection [31, 38]. ICD via heat treatment is validated in domestic dogs [27] and has been shown to increase antigen detection in cats, which are atypical hosts for *D. immitis* [30]. Antigen testing following ICD was able to identify infection in an Asian small-clawed otter in a sample from 2018 that was negative on the same antigen test performed prior to ICD (Table 8). Interestingly, a sample from the same otter in 2021 was positive on both the pre-ICD and post-ICD antigen testing, indicating potential disease progression. Both these animals were reported to be receiving cattle ivermectin orally as monthly heartworm prevention and were undergoing routine testing every one to three years via the feline antigen and feline antibody tests, via the study questionnaire. During a follow-up with the facility, however, the positive antigen detection was not known. These results suggest that ICD may be used in mustelids in addition to canids and felids to identify false-negative antigen tests and may help zoos in identifying more cases of *D. immitis* if adopted as part of routine testing strategies.

## Conclusion

The absence of labeled prevention products for exotic and wildlife species and the lack of information regarding effective preventive doses of off-label products, along with ethical and logistical limitations in conducting research on captive wildlife and endangered species, serve as barriers to effective prevention strategies. However, despite the endemicity of heartworm disease in Texas and the relatively high perceived risk of heartworm disease, the proportion of antigen detection in this study was low. This study may serve as a baseline for future studies assessing the prevalence of *D. immitis* in zoo facilities in heartworm-endemic areas of the United States, where resistance to ML drugs is an emerging concern.

## Supplementary Information

**Additional file 1: Table S1.** Sample distribution and pre- and post-ICD antigen test results by carnivore family.

## Data Availability

All data are presented in the manuscript, figures, and additional tables. Further inquiries may be directed to the corresponding author.

## Abbreviations

AHS: American Heartworm Society
AZA: Association of Zoos and Aquariums
CAPC: Companion Animal Parasite Council
ICD: Immune complex dissociation
ML: Macrocyclic lactone
PCI_2_: Potential for Conflict Index, second generation
ZAA: Zoological Association of America

## References

[1] McCall JW, Genchi C, Kramer LH, Guerrero J, Venco L. Heartworm disease in animals and humans. Adv Parasitol. 2008;66:193–285. 10.1016/S0065-308X(08)00204-2 18486691

[2] Bowman DD, Atkins CE (2009). Heartworm biology, treatment, and control. Vet Clin North Am Small Anim Pract.

[3] Drake J, Wiseman S (2018). Increasing incidence of *Dirofilaria immitis* in dogs in USA with focus on the southeast region 2013–2016. Parasit Vectors.

[4] Nelson CT, McCall JW, Jones S, Moorhead A. Current canine guidelines for the prevention, diagnosis, and management of the heartworm (*Dirofilaria immitis*) infection in dogs. Wilmington (Ed.). https://www.heartwormsociety.org/veterinary-resources/ American Heartworm Society; 2020.

[5] Thornton JE, Bell RR, Reardon MJ (1974). Internal parasites of coyotes in southern Texas. J Wildl Dis.

[6] Kazacos KR, Edberg EO (1979). *Dirofilaria immitis* infection in foxes and coyotes in Indiana. J Am Vet Med Assoc.

[7] Paras KL, Little SE, Reichard MV, Reiskind MH (2012). Detection of *Dirofilaria immitis* and *Erlichia* species in coyotes (*Canis latrans*), from rural Oklahom and Texas. J Vector Borne Dis.

[8] Penezic A, Selakovic S, Pavlovic I, Cirovic D (2014). First findings and prevalence of adult heartworms (*Dirofilaria immitis*) in wild carnivores from Serbia. Parasitol Res.

[9] Aher AM, Caudill D, Caudill G, Butryn RS, Wolf D, Fox M (2016). Prevalence, genetic analyses, and risk factors associated with heartworm (*Dirofilaria immitis*) in wild coyotes (*Canis latrans*) from Florida, USA. J Wild Dis.

[10] Jean St, Bryan LK, Sobotyk C, Verocai GG (2022). Pathology in Practice. J Am Vet Med A.

[11] Acosta L, León-Quinto T, Bornay-Llinares FJ, Simón MÁ, Simón F, Morchón R (2019). *Dirofilaria immitis*: a new potential pathogen for the endangered Iberian lynx (*Lynx pardinus*). Intern J Appl Res Vet Med.

[12] Pence DB, Tewes ME, Laack LL (2003). Helminths of the ocelot from southern Texas. J Wildl Dis.

[13] Filoni C, Pena HF, Gennari SM, Cristo DS, Torres LN, Catão-Dias JL (2009). Heartworm (*Dirofilaria immitis*) disease in a Brazilian oncilla (*Leopardus tigrinus*). Pesq Vet Bras.

[14] Murata K, Yanai T, Agatsuma T, Uni S (2003). *Dirofilaria immitis* infection of a snow leopard (*Uncia uncia*) in a Japanese zoo with mitochondrial DNA analysis. J Vet Med Sci.

[15] Mazzariol S, Cassini R, Voltan L, Aresu L, Frangipane di Regalbono A (2010). Heartworm (*Dirofilaria immitis*) infection in a leopard (*Panthera pardus pardus*) housed in a zoological park in north-eastern Italy. Parasit Vectors.

[16] Okada R, Imai S, Ishii T (1983). Clouded leopard, *Neofelis nebulosa*, new host for *Dirofilaria immitis*. Japanese J Vet Sci.

[17] Ruiz de Ybanez MR, Martinez-Carrasco C, Martinez JJ, Ortiz JM, Attout T, Bain O (2006). *Dirofilaria immitis* in an African lion (*Panthera leo*). Vet Rec.

[18] Deem SL, Heard DJ, LaRock R (1998). Heartworm (*Dirofilaria immitis*) disease and glomerulonephritis in a black-footed cat (*Felis nigripes*). J Zoo Wildl Med.

[19] Snyder DE, Hamir AN, Hanlon CA, Rupprecht CE (1989). *Dirofilaria immitis* in a raccoon (*Procyon lotor*). J Wildl Dis.

[20] Snyder DE, Hamir AN, Nettles VF, Rupprecht CE (1989). *Dirofilaria immitis* in a river otter (*Lutra canadensis*) from Louisiana. J Wildl Dis.

[21] Penezic A, Moriano R, Spasic M, Cirovic D (2018). First report of a naturally patent infection with *Dirofilaria immitis* in an otter (*Lutra lutra*). Parasitol Res.

[22] Upton KE, Sobotyk C, Edwards EE, Verocai GG (2022). *Dirofilaria immitis* in an Asian small-clawed otter (*Aonyx cinereus*) from southeastern Louisiana, United States. Vet Parasitol: Reg Stud Rep.

[23] Crum JM, Nettles VF, Davidson WR (1978). Studies on endoparasites of the black bear (*Ursus americanus*) in the southeastern United States. J Wildl Dis.

[24] Papadopoulos E, Komnenou A, Poutachides T, Heikkinen P, Oksanen A, Karamanlidis AA (2017). Detection of *Dirofilaria immitis* in a brown bear (*Ursus arctos*) in Greece. Helminthologia.

[25] Self SW, Pulaski CN, McMahan CS, Brown DA, Yabsley MJ, Gettings JR (2019). Regional and local temporal trends in the prevalence of canine heartworm infection in the contiguous United States: 2012–2018. Parasit Vectors.

[26] Companion Animal Parasite Council, 2021. Canine parasite prevalence maps (United States) https://capcvet.org 2021.

[27] Beall MJ, Arguello-Marin A, Drexel J, Liu J, Chandrashekar R, Alleman AR (2017). Validation of immune complex dissociation methods for use with heartworm antigen tests. Parasit Vectors.

[28] Weil GJ, Malane MS, Powers KG, Blair LS (1985). Monoclonal antibodies to parasite antigens found in the serum of *Dirofilaria immitis*-infected dogs. J Immunol.

[29] Vaske JJ, Beaman J, Barreto H, Shelby LB (2010). An extension and further validation of the Potential for Conflict Index. Leis Sci.

[30] Gruntmeir JM, Adolph CB, Thomas JE, Reichard MV, Blagburn BL, Little SE (2017). Increased detection of *Dirofilaria immitis* antigen in cats after heat pretreatment of samples. J Feline Med Surg.

[31] Gruntmeir JM, Long MT, Blagburn BL, Walden HS (2020). Canine heartworm and heat treatment: an evaluation using a well based enzyme-linked immunosorbent assay (ELISA) and canine sera with confirmed heartworm infection status. Vet Parasitol.

[32] AZA Jaguar Species Survival Plan (2016). Jaguar Care Manual Association of Zoos and Aquariums.

[33] AZA Lion Species Survival Plan (2012). Lion Care Manual Association of Zoos and Aquariums.

[34] AZA Canid TAG (2012). Large Canid (Canidae) Care Manual Association of Zoos and Aquariums.

[35] Longhofer SL, Daurio CP, Plue RE, Alva R, Wallace DH, Roncalli RA. Ivermectin for the prevention of feline heartworm disease. In: Proceedings of the Heartworm Symposium ’95, Auburn, AL. American Heartworm Society. 1995;177–182.

[36] Lobetti RG, Caldwell P (2012). Doramectin toxicity in a group of lions (*Panthera leo*): clinical communication. J S Afr Vet Assoc.

[37] Hodo CL, Rodriguez JY, Curtis-Robles R, Zecca IB, Snowden KF, Cummings KJ (2019). Repeated cross-sectional study of *Trypanosoma cruzi* in shelter dogs in Texas, in the context of *Dirofilaria immitis* and tick-borne pathogen prevalence. J Vet Intern Med.

[38] Hayes KM, Rodriguez JY, Little SE, Litster AL, Mwacalimba KK, Sundstrom KD (2020). Heartworm prevalence in dogs versus cats: multiple diagnostic modalities provide new insights. Vet Parasitol.

[39] Custer JW, Pence DB (1981). Dirofilariasis in wild canids from the Gulf coastal prairies of Texas and Louisiana, USA. Vet Parasitol.

[40] Sobotyk C, Nguyen N, Negron V, Varner A, Saleh M, Hilton C (2022). Detection of *Dirofilaria immitis* via integrated serological and molecular analyses in coyotes from Texas, United States. Int J Parasitol Parasites Wildl.

[41] Lan J, Fu Y, Yang Z, Zhang Z, Wang C, Luo L (2012). Treatment and prevention of natural heartworm (*Dirofilaria immitis*) infections in red pandas (*Ailurus fulgens*) with selamectin and ivermectin. Parasitol Int.

[42] Jara RF, Wydeven AP, Samuel MD (2016). Gray wolf exposure to emerging vector-borne diseases in Wisconsin with comparison to domestic dogs and humans. Plos ONE.

[43] Smout FA, Skerratt LF, Butler JRA, Johnson CN, Congdon BC (2016). Dingoes (*Canis dingo* Meyer, 1793) continue to be an important reservoir host of *Dirofilaria immitis* in low density housing areas in Australia. Vet Parasitol.

[44] Orozco MM, Ceballos LA, de la Cruz PM, Gurtler RE (2014). Local threats and potential infectious hazards to maned wolves (*Chrysocyon brachyurus*) in the southeastern Argentine Chaco. Mammalia.

[45] King AW, Bohning AM (1984). The incidence of heartworm *Dirofilaria immitis* (Filarioidea), in the wild canids of northeast Arkansas. Southwest Nat.

[46] Otto GF (1974). Occurrence of the heartworm in unusual locations and in unusual hosts. Am Heartworm Soc Symp Proc.

[47] Kennedy S, Patton S (1981). Heartworms in a Bengal tiger (*Panthera tigris*). J Zoo Wildl Med.

[48] Helmick KE, Koplos P, Raymond J (2006). Disseminated coccidioidomycosis in a captive Indochinese tiger (*Panthera tigris corbetti*) with chronic renal disease. J Zoo Wildl Med.

[49] Foley JE, Swift P, Fleer KA, Torres S, Girard YA, Johnson CK (2013). Risk factors for exposure to feline pathogens in California mountain lions (*Puma concolor*). J Wildl Dis.

[50] McHale B, Callahan RT, Paras KL, Weber M, Kimbrell L, Brock P (2020). Sparganosis due to *Spirometra* sp. (Cestoda; Diphyllobothriidae) in captive meerkats (*Suricata suricatta*). Int J Parasitol Parasites Wildl.

