# Supplementary material for: Heartworm (Dirofilaria immitis) in carnivores kept in zoos in Texas, USA: risk perception, practices, and antigen detection
Source: Parasit Vectors. 2023 Apr 28;16:150. doi: 10.1186/s13071-023-05750-z (PMC10142401; doi:10.1186/s13071-023-05750-z)
Supplement: Supplementary file 1 — Additional file 1: Table S1. Sample distribution and pre- and post-ICD antigen test results by carnivore family. [file 13071_2023_5750_MOESM1_ESM.docx]

Table S1-Sample Distribution and Pre- and Post-ICD Antigen Test Results by Carnivore Family

| **Family and Species** | **Common name of species** | **# Samples** | **Facility** | **Pre-ICD** | **Post-ICD** |
| --- | --- | --- | --- | --- | --- |
| Ailuridae |  | 2 |  |  |  |
| *Ailurus fulgens* | Red Panda | 2 | E | Negative | Negative |
|  |  |  | E | Negative | Negative |
| Canidae |  | 37 |  |  |  |
| *Canis latrans*# | Coyote | 1 | D | Negative | Negative |
| *Canis lupus baileyi* | Mexican Gray Wolf | 5 | A | Negative | Negative |
|  |  |  | A | Negative | Negative |
|  |  |  | A | Negative | Negative |
|  |  |  | A | Negative | Negative |
|  |  |  | A | Negative | Negative |
| *Canis lupus dingo* | Dingo | 2 | E | Negative | Negative |
| # |  |  | E | Negative | Negative |
| *Canis rufus* | Red Wolf | 3 | A | Negative | Negative |
|  |  |  | A | Negative | Negative |
|  |  |  | A | Negative | Negative |
| *Chrysocyon brachyurus* | Maned Wolf | 7 | A | Negative | Negative |
|  |  |  | A | Negative | Negative |
|  |  |  | A | Negative | Negative |
|  |  |  | A | Negative | Negative |
|  |  |  | A | Negative | Negative |
|  |  |  | A | Negative | Negative |
|  |  |  | E | Negative | Negative |
| *Lycaon pictus* | African Painted Dog | 10 | B | Negative | Negative |
|  |  |  | B | Negative | Negative |
|  |  |  | B | Negative | Negative |
|  |  |  | E | Negative | Negative |
|  |  |  | E | Negative | Negative |
|  |  |  | E | Negative | Negative |
|  |  |  | E | Negative | Negative |
|  |  |  | E | Negative | Negative |
|  |  |  | D | Negative | Negative |
|  |  |  | D | Negative | Negative |
| *Otocyon megalotis megalottis* | Bat-eared Fox | 1 | B | Negative | Negative |
| *Speothos venaticus* | Bush Dog | 3 | B | Negative | Negative |
|  |  |  | B | Negative | Negative |
|  |  |  | B | Negative | Negative |
| *Urocyon cinereoargenteus* | Grey Fox | 1 | D | Negative | Negative |
| *Vulpes velox* | Swift Fox | 3 | E | Negative | Negative |
| # |  |  | D | Negative | Negative |
|  |  |  | D | Negative | Negative |
| *Vulpes zerda* | Fennec Fox | 1 | C | Negative | Negative |
| Eupleridae |  | 3 |  |  |  |
| *Cryptoprocta ferox* | Fossa | 3 | B | Negative | Negative |
|  |  |  | E | Negative | Negative |
|  |  |  | E | Negative | Negative |
| Felidae |  | 113 |  |  |  |
| *Acinonyx jubatus* | Cheetah | 53 | A | Negative | Negative |
|  |  |  | A | Negative | Negative |
|  |  |  | A | Negative | Negative |
|  |  |  | A | Negative | Negative |
|  |  |  | A | Negative | Negative |
|  |  |  | A | Negative | Negative |
|  |  |  | A | Negative | Negative |
|  |  |  | A | Negative | Negative |
|  |  |  | A | Negative | Negative |
|  |  |  | A | Negative | Negative |
|  |  |  | A | Negative | Negative |
|  |  |  | A | Negative | Negative |
|  |  |  | A | Negative | Negative |
|  |  |  | A | Negative | Negative |
|  |  |  | A | Negative | Negative |
|  |  |  | A | Negative | Negative |
|  |  |  | A | Negative | Negative |
|  |  |  | A | Negative | Negative |
|  |  |  | A | Negative | Negative |
|  |  |  | A | Negative | Negative |
|  |  |  | A | Negative | Negative |
|  |  |  | A | Negative | Negative |
|  |  |  | A | Negative | Negative |
|  |  |  | A | Negative | Negative |
|  |  |  | A | Negative | Negative |
|  |  |  | A | Negative | Negative |
|  |  |  | A | Negative | Negative |
|  |  |  | A | Negative | Negative |
|  |  |  | A | Negative | Negative |
|  |  |  | A | Negative | Negative |
|  |  |  | A | Negative | Negative |
|  |  |  | A | Negative | Negative |
|  |  |  | A | Negative | Negative |
|  |  |  | A | Negative | Negative |
| Duplicate sample |  |  | A | Negative | Negative |
| Duplicate sample |  |  | A | Negative | Negative |
|  |  |  | A | Negative | Negative |
|  |  |  | A | Negative | Negative |
|  |  |  | A | Negative | Negative |
|  |  |  | A | Negative | Negative |
|  |  |  | A | Negative | Negative |
|  |  |  | A | Negative | Negative |
|  |  |  | A | Negative | Negative |
| * |  |  | B | Negative | Negative |
| * |  |  | E | Negative | Negative |
| * |  |  | E | Negative | Negative |
| * |  |  | E | Negative | Negative |
| * |  |  | E | Negative | Negative |
| # |  |  | D | Negative | Negative |
|  |  |  | D | Negative | Negative |
|  |  |  | D | Negative | Negative |
| * |  |  | C | Negative | Negative |
| * |  |  | C | Negative | Negative |
| *Caracal caracal** | Caracal | 3 | B | Negative | Negative |
| * |  |  | C | Negative | Negative |
| * |  |  | C | Negative | Negative |
| *Leopardus pardalis** | Ocelot | 5 | B | Negative | Negative |
| * |  |  | E | Negative | Negative |
| * |  |  | E | Negative | Negative |
|  |  |  | D | Negative | Negative |
| * |  |  | C | Negative | Negative |
| *Leptailurus serval* | Serval | 1 | D | Negative | Negative |
| *Lynx rufus** | Bobcat | 3 | E | Negative | Negative |
|  |  |  | D | Negative | Negative |
|  |  |  | D | Negative | Negative |
| *Neofelis nebulosa** | Clouded Leopard | 5 | B | Negative | Negative |
| * |  |  | E | Negative | Negative |
| * |  |  | E | Negative | Negative |
| * |  |  | E | Negative | Negative |
| * |  |  | E | Negative | Negative |
| *Panthera leo krugeri** | African Lion | 15 | B | Negative | Negative |
| * |  |  | B | Negative | Negative |
| * |  |  | B | Negative | Negative |
| * |  |  | E | Negative | Negative |
| * |  |  | E | Negative | Negative |
| * |  |  | E | Negative | Negative |
| * |  |  | E | Negative | Negative |
| * |  |  | E | Negative | Negative |
|  |  |  | D | Negative | Negative |
|  |  |  | D | Negative | Negative |
|  |  |  | D | Negative | Negative |
|  |  |  | D | Negative | Negative |
| # |  |  | D | Negative | Negative |
|  |  |  | D | Negative | Negative |
| * |  |  | C | Negative | Negative |
| *Panthera onca** | Jaguar | 10 | B | Negative | Negative |
| * |  |  | B | Negative | Negative |
| * |  |  | E | Negative | Negative |
| * |  |  | E | Negative | Negative |
| * |  |  | E | Negative | Negative |
| *# |  |  | E | Negative | Negative |
| * |  |  | E | Negative | Negative |
|  |  |  | D | Negative | Negative |
|  |  |  | D | Negative | Negative |
| # |  |  | D | Negative | Negative |
| *Panthera pardus** | Leopard | 1 | E | Negative | Negative |
| *Panthera tigris* | White Tiger | 1 | D | Negative | Negative |
| *Panthera tigris jacksoni** | Malayan Tiger | 5 | E | Negative | Negative |
| * |  |  | E | Negative | Negative |
|  |  |  | D | Negative | Negative |
|  |  |  | D | Negative | Negative |
| * |  |  | C | Negative | Negative |
| *Panthera tigris sumatrae* | Sumatran Tiger | 7 | B | Negative | Negative |
| Duplicate sample* |  |  | C | Negative | Negative |
| Duplicate sample* |  |  | C | Negative | Negative |
| * |  |  | C | Negative | Negative |
| * |  |  | C | Negative | Negative |
| *# |  |  | C | Negative | Negative |
| * |  |  | C | Negative | Negative |
| *Prionailurus viverrinus** | Fishing Cat | 1 | B | Negative | Negative |
| *Puma concolor** | Cougar | 3 | E | Negative | Negative |
| * |  |  | E | Negative | Negative |
| * |  |  | E | Negative | Negative |
| Herpestidae |  | 17 |  |  |  |
| *Helogale parvula* | Common Dwarf Mongoose | 1 | B | Negative | Negative |
| *Mungos mungo* | Banded Mongoose | 4 | E | Negative | Negative |
|  |  |  | E | Negative | Negative |
|  |  |  | E | Negative | Negative |
|  |  |  | E | Negative | Negative |
| *Suricata suricatta* | Slender-tailed Meercat | 12 | E | Negative | Negative |
|  |  |  | E | Negative | Negative |
|  |  |  | E | Negative | Negative |
|  |  |  | E | Negative | Negative |
| # |  |  | E | Negative | Negative |
|  |  |  | E | Negative | Negative |
|  |  |  | E | Negative | Negative |
|  |  |  | E | Negative | Negative |
|  |  |  | E | Negative | Negative |
|  |  |  | E | Negative | Negative |
|  |  |  | E | Negative | Negative |
|  |  |  | C | Negative | Negative |
| Hyaenidae |  | 5 |  |  |  |
| *Crocuta crocuta* | Spotted Hyena | 2 | B | Negative | Negative |
|  |  |  | B | Negative | Negative |
| *Hyaena hyaena* | Striped Hyena | 3 | D | Negative | Negative |
|  |  |  | D | Negative | Negative |
|  |  |  | D | Negative | Negative |
| Mephitidae |  | 2 |  |  |  |
| *Mephitis mephitis*# | Striped Skunk | 2 | B | Negative | Negative |
|  |  |  | E | Negative | Negative |
| Mustelidae |  | 17 |  |  |  |
| *Aonyx cinereus* | Asian Small-clawed Otter | 12 | B | Negative | Negative |
|  |  |  | B | Negative | Negative |
|  |  |  | E | Negative | Negative |
|  |  |  | E | Negative | Negative |
| Duplicate sample* |  |  | C | Negative | Positive |
| Duplicate sample*# |  |  | C | Positive | Positive |
| # |  |  | C | Negative | Negative |
| Triplicate sample |  |  | C | Negative | Negative |
| Triplicate sample |  |  | C | Negative | Negative |
| Triplicate sample# |  |  | C | Negative | Negative |
| Duplicate sample* |  |  | C | Positive | Positive |
| Duplicate sample* |  |  | C | Positive | Positive |
| *Lontra canadensis* | North American River Otter | 5 | E | Negative | Negative |
|  |  |  | E | Negative | Negative |
|  |  |  | D | Negative | Negative |
|  |  |  | D | Negative | Negative |
|  |  |  | F | Negative | Negative |
| Prionodontidae |  | 0 |  |  |  |
| Procyonidae |  | 14 |  |  |  |
| *Bassariscus astutus* | Ringtail | 5 | B | Negative | Negative |
|  |  |  | B | Negative | Negative |
|  |  |  | B | Negative | Negative |
|  |  |  | D | Negative | Negative |
|  |  |  | D | Negative | Negative |
| *Nasua narica* | White-nosed Coati | 5 | E | Negative | Negative |
|  |  |  | E | Negative | Negative |
|  |  |  | D | Negative | Negative |
|  |  |  | D | Negative | Negative |
|  |  |  | D | Negative | Negative |
| *Potos flavus* | Kinkajou | 2 | B | Negative | Negative |
|  |  |  | E | Negative | Negative |
| *Procyon lotor* | Racoon | 2 | E | Negative | Negative |
|  |  |  | E | Negative | Negative |
| Ursidae |  | 6 |  |  |  |
| *Tremarctos ornatus* | Spectacled Bear | 2 | B | Negative | Negative |
|  |  |  | E | Negative | Negative |
| *Ursus americanus* | American Black Bear | 3 | B | Negative | Negative |
|  |  |  | E | Negative | Negative |
|  |  |  | E | Negative | Negative |
| *Ursus americanus luteolus* | Louisiana Black Bear | 1 | D | Negative | Negative |
| Viverridae |  | 1 |  |  |  |
| *Arctictis binturong* | Binturong | 1 | E | Negative | Negative |

*Feline antibody tested

#EDTA diluted Sample
